# How do Indonesian youth perceive cigarette advertising? A cross-sectional study among Indonesian high school students

**DOI:** 10.3402/gha.v9.30914

**Published:** 2016-08-29

**Authors:** Yayi Suryo Prabandari, Arika Dewi

**Affiliations:** 1 Department of Health Behavior, Environment Health & Social Medicine, Faculty of Medicine, Universitas GadjahMada, Yogyakarta, Indonesia; 2 Quit Tobacco Indonesia, Center for Health Behavior and Promotion, Faculty of Medicine, Universitas Gadjah Mada, Yogyakarta, Indonesia

**Keywords:** perception, cigarette ads, smoking status, Indonesia

## Abstract

**Background:**

Previous studies have reported an association between cigarette advertising and smoking behavior. Although this has been reported extensively in the West, it has been reported less in Southeast Asian countries that have not completely banned tobacco advertising promotion and sponsorship (TAPS). Indonesia is the only ASEAN country that has not ratified the Framework Convention on Tobacco Control, so TAPS regulation is limited. This study aimed to assess the association between youths’ perceptions of cigarette ads and smoking initiation.

**Design:**

We conducted a cross-sectional survey among 2,115 high school students aged 13–18 years in Yogyakarta, Indonesia. A self-administered questionnaire was distributed to gauge the perception of cigarette ads and initiation to smoking. We calculated the odds ratio (OR) between the perception of cigarette ads and smoking initiation, adjusting for sociodemographic and psychosocial variables. The sociodemographic variables included in the final model were age and sex.

**Results:**

The final multivariate model showed an association between perception of tobacco ads encouraging youths to smoke and smoking initiation (OR 2.70) and current smoking (OR 7.63). Attitude toward TAPS was associated with smoking initiation (OR 1.51) and current smoking (OR 3.32). Exposure to cigarette ads had an association with smoking initiation only (OR 1.27) and did not have an association with current smoking. Having friends and family who smoked was associated with smoking initiation and current smoking in the final multivariate model. Smoking initiation and current smoking were also related to the susceptibility to smoke.

**Conclusions:**

This study revealed that cigarette ads were perceived as encouraging youths to smoke and that smoking status was consistently associated with perception of cigarette ads targeted at youths, attitude toward TAPS, and susceptibility as well as smoking friends and family. Regulations to ban TAPS, particularly cigarette ads for preventing youths from smoking, should be adopted rapidly in Indonesia, where tobacco control remains limited.

## Introduction

As reported in the *Tobacco Atlas* (1), the last three decades have seen a decrease in the prevalence of smokers aged 15 years and above in developed and developing countries. However, in some countries, including Indonesia, the prevalence of smokers in the general population has increased from 27.2% (2) in 1995 to 34.8% in 2011 (3) and 36.3% in 2013 (4). With a 67% prevalence of males aged 15 years (4) and above being smokers, Indonesia ranks third in the world for the number of male smokers and was number one in ASEAN countries in 2014 (5).

The number of smokers runs in parallel with Indonesia's position as ranking fifth in the world in cigarette production (6); more than 1,000 cigarette companies are based in Indonesia (7, 8). Consequently, the domination of the cigarette industry in Indonesia limits the ability of the country to control tobacco. Indonesia is the only Asia-Pacific country that has not ratified the Framework Convention on Tobacco Control (FCTC) (9), so tobacco advertising promotion and sponsorship (TAPS) still occurs. Cigarette advertisements are allowed on outdoor advertising and on printed and electronic media. The cigarette industry also sponsors many activities and scholarships. Although the industry claims that TAPS targets adults (10), the US Surgeon General's review in 2012 (11) reported that TAPS put adolescents at risk of smoking. Freedman et al. (12) reported that attending events sponsored by cigarette industries also risked promoting smoking; they stated that the cigarette industry's practice of advertising in magazines, cigarette sales, sponsorship, and their interactive media also focused attention on smoking (13).

The aggressiveness of TAPS in Indonesia over the years is in line with the increase of smoking prevalence among adolescents in Indonesia, as can be seen from the following pattern. The prevalence of smokers among school children (boys and girls) aged 15–19 years in 1986 was 13.2%, which increased to 22.6% in 1995 (14). Riskesdas or Basic Health Survey 2007 (15), 2010 (16), and 2013 (4) data showed the smoking prevalence of individuals aged 15–24 years among both genders was 24.5, 26.6, and 26.2% respectively. For male smokers aged 15–19 years, the prevalence was 16% in 1995 (2), 51.70% in 2011 (17), and 74.4% in 2013 (18).

The increasing prevalence of smokers, particularly among youths, has not been balanced by health-promotional activities or tobacco control. Tobacco regulation in Indonesia was very limited before the 1990s (19). Although cigarette advertising in electronic media has not existed for long, there has been a tremendous amount of outdoor cigarette advertising because of the decentralization policies existing in Indonesia since the beginning of 2000 (20). In 2007, as many as 99.7% of youths in Indonesia reported seeing tobacco advertisements on television, 87% on billboards, and 76% in print media; and 81% had attended at least one event sponsored by the tobacco industry in their lifetime (21). The *Tobacco Atlas* also reported that there were 42 countries in where more than 70% of youths (13–15 years) noticed tobacco advertising on billboards during the last 30 days, whereas in Indonesia the percentage was 89% (1).

TAPS influences adolescents to smoke in many ways, and a number of reviews have addressed the relationship of TAPS with adolescent's behavior. Henriksen et al. (22) stated that the way cigarettes are sold and the point of sale have a relationship with smoking behavior. They followed adolescents for a year and asked them how many times they went to stores that sold cigarettes and how many times they saw cigarette advertisements in those stores. Morgenstern et al. (23) investigated the relationship between perception of cigarette advertisement and smoking initiation. Gendall et al. (24) and Saebo and Lund (25) reported that cigarette exposure in movies was a potential risk that was related to adolescents smoking; however, these studies were carried out in New Zealand and Norway, where tobacco control is strict. Freeman (12) investigated adolescent's impression of cigarette advertisements that were addressed to youth, with smoking behavior. Cigarette and anti-cigarette advertisement exposure was researched by Abdalla et al. in Saudi Arabia, which revealed that the non-smokers were more frequently exposed to anti-cigarette advertisement, whereas smokers were more frequently exposed to cigarette advertisement (26).

Smoking initiation in adolescents is influenced by many factors besides TAPS. Previous research has shown that adolescents start to smoke because of their parents, relatives, and friends who smoke (27, 28). The confounding factors were sex, level of education, type of school (general or vocational), perceived academic performance, perceived stress level, frequency of alcohol drinking per month, and whether they purchase cigarettes for their parents or someone else (29). Madkour et al. (30) found that among boys, cigarette advertising and promotion were significantly associated with their current smoking status, whereas among both boy and girl groups, they were positively associated with initiation susceptibility. Dahal et al. (31) included sociodemographic variables (sex, perceived educational status, and pocket money) in addition to smoking and alcohol use by a family member and found that cigarette smoking status had significant relationship with media-related variables like seeing cigarette advertisement, attending cigarette companies sponsored musical program, and so on. Hanewinkel et al. (32) found that compared with low exposure to cigarette advertisements, high exposure remained a significant predictor of adolescent smoking initiation after controlling for the covariates of age, gender, socioeconomic status (SES), rebelliousness, and sensation seeking as covariate variables beside family and friends who smoke. Shadel and Cervone (33) investigated the relationship of an individual's self-concept and ad exposure with smoking intention and showed that adolescents with high self-control and low internal conflicts were more likely not to smoke even though they were exposed to TAPS. Fulmer et al. (34) and Hanewinkel et al. (35) revealed that odds ratio (OR) of susceptibility to smoking was higher among adolescents who were exposed to cigarette ads. Yang et al. (36) in the research of other individual factors found that perceived prevalence of peer smoking was the intermediate factor between media exposure and smoking intention.

In a country like Indonesia, where TAPS is aggressive and tobacco control is not strict, information is needed on whether youths perceive cigarette ads as being related to smoking initiation or whether smoking initiation is related to other variables, such as social influence. The Yogyakarta Special Region (YSR) was selected as the study site because large number of young people come to YSR each year to pursue their studies, not only for tertiary degrees but also for primary and secondary education. Along with the introduction of decentralization policies in 2000, the government of Yogyakarta allowed industries, including the cigarette industry, to advertise through outdoor media, including billboards, banners, posters, and neon boxes, to increase tax revenue from the provinces. This has resulted in extensive cigarette advertising in many places in Yogyakarta. The present study was designed to expand previous research on the association between the perception of cigarette ads, exposure to cigarette ads, and marketing with smoking initiation.

## Methods

### Study procedure

We conducted a cross-sectional study in Yogyakarta Municipality in 2010. Yogyakarta Municipality is one of five districts in the YSR, located in the center of the Island of Java. The YSR has the second highest population density of any province in Indonesia, after Jakarta. The proportion of the population in the younger age groups is large, with those aged 10–14 and 15–19 years comprising 7.6 and 10% of the total population, respectively.

Students were selected from a list of high schools issued by the Department of Education and Culture, Yogyakarta Municipality, using a multistage sampling scheme. We divided the schools into two categories – public and private – and then randomly chose the schools. We then used random selection to choose a class, and all students in the class participated in this study. Self-reported questionnaires were distributed to 2,115 male and female students. Research assistants went to classes in each selected school and explained the procedure for completing the questionnaire. All students gave their consent, and parents or legal guardians gave informed consent. Ethics approval was granted by the Medical and Health Research Ethics Committee, Faculty of Medicine, Universitas Gadjah Mada – Dr. Sardjito General Hospital, and permission was obtained from the YSR Planning Agency as well as the Yogyakarta Municipality Department of Education and Culture.

### Variables

#### Dependent variables

##### Smoking initiation

We assessed each participant's smoking initiation by asking the following: ‘Have you ever smoked, even just a puff?’ and ‘How do you describe your recent smoking practice?’ We classified participants who reported that they had smoked, even just a puff, or had tried to smoke a few times with friends or never smoked again after the first puff as participants who had initiated smoking. Participants who reported that they had never smoked, even just a puff, were reported as not having initiated smoking. We adopted the smoking initiation definition from Freedman (12) and Henriksen et al. (22) (‘Initiation is a phenomenon of smoking onset or the progression from non-smoker to experimental smoker’) and Mayhew et al. (37) (‘Initiation is the tried stage in which somebody tried to puff one or two cigarettes’).

##### Smoking status

Participants were classified as current smokers if they reported that they had smoked at least one cigarette in the 7 days preceding the study (adopted from Andrews et al. (38)) when responding to the question, ‘Have you smoked at least one cigarette in the last seven days?’ and/or reported they had sometimes smoked or smoked every day when responding to the question, ‘How do you describe your recent smoking practice?’ All others were classified as non-smokers.

#### Independent variables

##### Perception of cigarette advertisement targeted at youths

We assessed participants’ perceptions of cigarette advertisement targeted at youths by asking the question, ‘What cigarette advertisement, based on your opinion, is targeted at youths?’ The options for the questions were ‘yes’, ‘no’, or ‘do not know’ for 15 cigarette advertisements, which were selected based on a previous study (39). The 15 cigarette advertisements were scored from 1 to 4 if participants responded ‘yes’ on each cigarette advertisement. The weighting of the score was based on previous results (39) that reported demographic targets of cigarette advertising. Score 4 was given for responding to cigarette advertisements of Star Mild, Class Mild, Marlboro Filter, and Djarum Super Kretek Filter, which targeted adolescents (age 12–18 years); score 3 was given for responding to cigarette advertisements of Lucky Strike Filter, Djarum Black, LA Light Kretek Filter, and Gudang Garam Kretek Filter International, which targeted young adults (age 19–25 years); score 2 was given for responding to cigarette advertisements of Dji Sam Soe Kretek and Sampoerna A Mild, which targeted adults (age 26–35 years), and score 1 was given for responding to cigarette advertisements of Dji Sam Soe Filter, Djarum 76, Gudang Garam Kretek Filter Professional, and Wismilak (Diplomat), which targeted older adults (age 36–60 years). Score 0 was given to any participant responding ‘no’ or ‘do not know’. Participants were classified as ‘high’ for perceiving cigarette advertisement as targeted at youths if the total score was 1 or more and ‘low’ if the total score was 0.

##### Perception of cigarette advertisement encouraging youths to smoke

The question of assessing this variable was ‘Which one of the cigarette advertisements shown below encourages you to smoke?’ The options for the questions were ‘yes’, ‘no’, or ‘do not know’ on 15 selected cigarette advertisements, similar to the previous variable. The score weighting was also similar to the previous variable. Participants were classified as ‘high’ on perception of cigarette advertisement encouraging youths to smoke if the total score was 1 or more and ‘low’ if the total score was 0.

##### Perception of cigarette advertisement message

We asked the question, ‘What is your impression when seeing cigarette advertisements?’ to measure the perception of 11 cigarette advertisement messages of individual themes (masculinity, friendship, attractive, mature, popular, enjoyment, being a modern woman), social values (nationalism), and the positive impact of smoking (creativity, concentration, and stress reduction). The themes were derived from previous research. Participants were classified as ‘high’ for perceiving a cigarette advertisement message if their score was 1 or more and ‘low’ if 0.

##### Attitude toward cigarette advertisements

To assess the attitude toward TAPS, we asked two questions: ‘In your opinion, do cigarette advertisements and sponsorship encourage youths to smoke?’ ‘Have you ever smoked due to the influence of a cigarette advertisement?’ Options for responding to the questions were ‘yes’ (score 1), ‘no’ (score 0), and ‘do not know’ (score 0). Participants were classified as having a positive attitude if they had a score of 2 and a negative attitude if they had a score of 1 or 0.

##### Exposure to cigarette advertisements

We assessed participants’ exposure to cigarette advertisements based on three questions. The first question was a proxy of ad recognition and was adopted from Hanewinkel et al. (35). First, ‘Have you ever seen a cigarette advertisement?’ (a picture of a cigarette advertisement with a women on it); second, ‘In your opinion, what is the cigarette message on that advertising?’; and third, ‘In your opinion, what was your impression of the woman on the general cigarette advertisement?’ Participants were classified as having had high exposure to cigarette advertisements if they reported they had seen the cigarette advertisement, enjoyed the message of the cigarette advertisement, or had any positive impression of the general cigarette advertisement (modern, stylish sexy, more freedom, adventure, and popular). Other options were classified as low exposure to cigarette advertisements.

##### Exposure to cigarette marketing

To assess the exposure to cigarette marketing, we asked two questions: ‘Do you have any cigarette merchandise?’ ‘Have you ever received free cigarettes from the tobacco industry?’ Participants were categorized as having had high exposure to cigarette marketing if they had had any cigarette merchandise or had ever received free cigarettes from the tobacco industry. Other responses were categorized as low exposure to cigarette marketing.

#### Covariate measurements

Covariate measures were proposed to control for confounding that would be theoretically related to TAPS exposure and smoking measures.

##### Sociodemographic variables

Gender (male and female), grade level (from grades 7 to 12 – junior and high school), pocket money (daily pocket money in IDR 1,000 [equal to US$ 0.20]), father's education (university, senior high school, junior high school, and elementary school – categorized as ‘university’ and ‘secondary’), mother's education (‘university’ and ‘secondary’).

##### Psychosocial variables

Friends smoke (0=none, 1=yes), family smokes (0=none, 1=one or more family members smoke), and exposure to tobacco control education (0=no, 1=yes). Other psychosocial variables were knowledge of tobacco harm and susceptibility to smoke. We assessed knowledge of the harm of tobacco based on three questions: ‘How many cigarette/s would be harmful to health?’ ‘Does second-hand smoke influence more than the smoker?’ and ‘Is the white cigarette more harmful than other cigarettes?’ Participants were categorized as having a high knowledge if they could correctly answer those three questions and were regarded as having low knowledge otherwise. The measurement of susceptibility to smoke was based on three questions: ‘Have you ever been challenged by your friends to smoke?’ ‘If you have ever been challenged by your friends to smoke, did you accept the challenge?’ ‘What was your reason for accepting the challenge?’ Participants were classified as susceptible if they were ever challenged to smoke or accepted the challenge because they were willing to try smoking. This question was modified from the Hanewinkel study (35).

### Data analysis

Out of 2,115 respondents, 1,943 completed the questionnaire. Two tables of analysis with OR and confidence interval (CI) values were derived from the data to assess perception, exposure, and attitude toward cigarette advertising and smoking initiation as well as the association with smoking status. We used univariate and multivariate logistic regression for examining the association between smoking initiation and smoking status with the perception of cigarette advertisement. Multivariate analysis was conducted to measure the best model to explain the association between perception, exposure, and attitude toward cigarette advertisement by adjusting sociodemographic and psychosocial variables. The selection of the best model was based on the parsimonious principle. Steps to build the model began with testing the association between cigarette advertising exposure, sociodemography, and social influence separately with outcome variables (smoking initiation and smoking status). We then combined all variables that had significant associations with smoking initiation and smoking status in the univariate analysis to build the model. A *p*-value of <0.05 was taken as significant. We used STATA version 13 for data analysis.

## Results

### Sociodemographic and psychosocial characteristics, smoking status, and perception of tobacco advertisement

More than half (52.50%) of the participants were female; one-third of the participants were from grade level 10, and more than half (51.52%) had a father with a university background, whereas the majority had a mother with a university or high school background (42.97%). As shown in Table 1, nearly 90% of the participants had a negative attitude toward cigarette advertising; one-third had a low level of cigarette advertisement exposure, and less than 10% of participants showed a high exposure to cigarette marketing and susceptibility to smoke. Furthermore, more than half of the participants reported that they highly perceived cigarette advertisement to be targeted at youths (55.79%), whereas 40% reported their perception on cigarette advertisement encouraging youths to smoke was low. Half of the participants had smoking friends or family, and their exposure to tobacco control education was somewhat high (71.69%). Participants’ knowledge of the harm caused by tobacco was high (66.65%).

**Table 1:** Characteristics of study participants

Variables			*n*	%
Demographic	Sex	Male	923	47.50
		Female	1,020	52.50
	Class	7	354	18.22
		8	306	15.75
		9	118	6.07
		10	640	32.94
		11	420	21.62
		12	105	5.40
	Pocket money (1,000 IDR)	7.000 IDR		
	Age (years)	15		
	Father education	University	1,001	51.52
		Senior HS	668	34.38
		Junior HS	127	6.54
		Elementary	147	7.57
	Mother education	University	835	42.97
		Senior HS	758	39.01
		Junior HS	169	8.70
		Elementary	181	9.32
Perceive on cigarette advertisement targeted to youth		Low	859	44.21
		High	1,084	55.79
Perceive on cigarette advertisement encouraging youth to smoke		Low	1,314	67.63
		High	629	32.37
Perceive on cigarette ads message		Low	846	43.54
		High	1,097	56.46
Attitude toward TAPS		Positive	212	10.91
		Negative	1,731	89.09
Exposure on cigarette ads		Low	1,287	66.24
		High	656	33.76
Exposure on cigarette marketing		Low	1,823	93.82
		High	120	6.18
Psychosocial	Friend smoke	No	935	48.12
		Yes	1,008	51.88
	Family smoke	No	983	48.12
		Yes	960	49.41
	Susceptibility to smoke	No	1,798	92.54
		Yes	145	7.46
	Knowledge of tobacco harm	Low	648	33.35
		High	1,295	66.65
	Exposure to tobacco control education	No	550	28.31
		Yes	1,393	71.69

### Univariate analysis of factors associated with smoking initiation

The perception of cigarette advertisement had a significant relationship with participants’ smoking initiation (Table 2). The odds of initiating smoking increased to 1.53 and 1.47, respectively, if participants had a high perception of cigarette advertisements being targeted at youths and of cigarette advertisement messages. The odds of initiating smoking tripled when participants were categorized as having a high perception of cigarette advertisement encouraging youths to smoke. Having a positive attitude toward cigarette advertisement (OR 3.74) and exposure to cigarette advertisement (OR 1.47) as well as exposure to cigarette marketing (OR 1.49) also was significantly related to smoking initiation. Participants who were susceptible to smoke were three times more likely to initiate smoking compared to those who were not susceptible to smoking. Furthermore, the risk of initiating smoking was three and a half times higher if participants had friends who smoked. However, exposure to tobacco control education and having good knowledge on the harm of tobacco had no relationship with smoking initiation. The sociodemographic variables that had a significant relationship with smoking initiation were gender, pocket money, and grade level (8, 10, and 11).

**Table 2:** Univariate analysis on smoking initiation

		Smoking initiation			
				
Variables	Category	No *n* (%)	Yes *n* (%)	OR (95% CI)	*p*
Sex	Female	868 (85.10)	152 (14.90)	1.00	
	Male	564 (61.11)	359 (38.89)	3.63 (2.93–4.52)	<0.001
Grade	7	285 (80.51)	69 (19.49)	1.00	
	8	213 (69.61)	93 (30.39)	1.80 (1.26–2.58)	0.001
	9	94 (79.66)	24 (20.34)	1.05 (0.63–1.77)	0.841
	10	449 (70.16)	191 (29.84)	1.76 (1.29–2.40)	<0.001
	11	301 (71.67)	119 (28.33)	1.63 (1.16–2.29)	0.004
	12	90 (85.71)	15 (14.29)	0.69 (0.38–1.26)	0.228
Age (years)		14.94	15.17	1.10 (1.03–1.18)	0.003
Pocket money (1,000 IDR)		6.93	7.62	1.03 (1.01–1.05)	0.008
Fathers education	University	748 (74.73)	253 (25.27)	1.00	
	Senior HS	491 (73.50)	177 (26.50)	1.06 (0.85–1.33)	0.576
	Junior HS	91 (71.65)	36 (28.35)	1.17 (0.78–1.76)	0.455
	Elementary	102 (69.39)	45 (30.61)	1.30 (0.89–1.19)	0.169
Mothers education	University	632 (75.69)	203 (24.31)	1.00	
	Senior HS	555 (73.22)	203 (26.78)	1.14 (0.91–1.43)	0.259
	Junior HS	119 (70.41)	50 (29.59)	1.31 (0.91–1.89)	0.151
	Elementary	126 (69.61)	55 (30.39)	1.36 (0.95–1.94)	0.089
Perception of cigarette advertisement targeted to youth	Low	672 (78.23)	187 (21.77)	1.00	
	High	760 (70.11)	324 (29.89)	1.53 (1.24–1.88)	<0.001
Perception of cigarette advertisement encouraging youth to smoke	Low	1,103 (83.94)	211 (16.06)	1.00	
	High	329 (52.31)	300 (47.69)	4.77 (3.85–5.91)	<0.001
Perception of cigarette ads message	Low	647 (76.48)	199 (23.52)	1.00	
	High	785 (71.56)	312 (28.44)	1.29 (1.05–1.59)	0.015
Attitude toward TAPS	Negative	1,332 (76.95)	399 (23.05)	1.00	
	Positive	100 (47.17)	112 (52.83)	3.74 (2.79–5.01)	<0.001
Exposure to cigarette ads	Low	982 (76.30)	305 (23.70)	1.00	
	High	450 (68.60)	206 (31.40)	1.47 (1.19–1.82)	<0.001
Exposure to cigarette marketing	Low	1.353 (74.22)	470 (25.78)	1.00	
	High	79 (65.83)	41 (34.17)	1.49 (1.01–2.21)	0.044
Friend smoke	No	799 (85.45)	136 (14.55)	1.00	
	Yes	633 (62.80)	375 (37.20)	3.48 (2.79–4.35)	<0.001
Family smoke	No	769 (78.23)	214 (21.77)	1.00	
	Yes	663 (69.06)	297 (30.94)	1.61 (1.31–1.97)	<0.001
Susceptibility to smoke	No	1,362 (75.75)	436 (24.25)	1.00	
	Yes	70 (48.28)	75 (51.72)	3.35 (2.37–4.72)	<0.001
Knowledge of tobacco harm	High	965 (74.52)	330 (25.48)	1.00	
	Low	467 (72.07)	181 (27.93)	1.13 (0.92–1.40)	0.248
Exposure to tobacco control education	No	421 (76.55)	129 (23.45)	1.00	
	Yes	1,011 (72.58)	382 (27.42)	1.23 (0.98–1.55)	0.074

### Univariate analysis of factors associated with smoking status

The pattern of the risk to become a smoker was almost similar with smoking initiation, but the odds were higher on becoming a smoker than the initiation to smoke. As presented in Table 3, participants who had a high perception of cigarette advertisement encouraging youths to smoke were 20 times more likely to become smokers compared to those who had a low perception. Participants with a high attitude toward cigarette advertisements were 10 times more likely to be smokers compared to those with a low attitude. Participants who were susceptible to smoke had odds of becoming a smoker that were 15 times that of those who were not susceptible to smoke. Moreover, participants whose friends were smokers were nine times more likely to become a smoker compared to those who had no exposure to this type of social influence.

**Table 3:** Univariate analysis on smoking status

		Smoking status			
				
Variables	Category	Non-smoker *n* (%)	Current smoker *n* (%)	OR (95% CI)	*p*
Sex	Female	960 (94.12)	60 (5.88)	1.00	
	Male	568 (61.54)	355 (38.46)	9.99 (7.46–13.40)	<0.001
Class	7	299 (84.46)	55 (15.54)	1.00	
	8	229 (74.84)	77 (25.16)	1.83 (1.24–2.69)	0.002
	9	95 (80.51)	23 (19.49)	1.32 (0.77–2.26)	0.317
	10	506 (79.06)	134 (20.94)	1.44 (1.02–2.03)	0.038
	11	307 (73.10)	113 (26.90)	2.00 (1.39–2.87)	<0.001
	12	92 (87.62)	13 (12.38)	0.77 (0.40–1.47)	0.425
Age		14.92	15.33	1.19 (1.11–1.28)	<0.001
Pocket money		6.93	7.79	1.03 (1.01–1.05)	0.002
Fathers education	University	825 (82.42)	176 (17.58)	1.00	
	Senior HS	504 (75.45)	164 (24.55)	1.53 (1.20–1.94)	0.001
	Junior HS	91 (71.65)	36 (28.35)	1.85 (1.21–2.82)	0.004
	Elementary	108 (73.47)	39 (26.53)	1.69 (1.13–2.53)	0.010
Mothers education	University	696 (83.35)	139 (16.65)	1.00	
	Senior HS	582 (76.78)	176 (23.22)	1.51 (1.18–1.94)	0.001
	Junior HS	120 (71.01)	49 (28.99)	2.04 (1.39–2.99)	<0.001
	Elementary	130 (71.82)	51 (28.18)	1.96 (1.35–2.85)	<0.001
Perception of cigarette advertisement targeted to youth	Low	732 (85.22)	127 (14.78)	1.00	
	High	796 (73.43)	288 (26.57)	2.09 (1.65–2.63)	<0.001
Perception of cigarette advertisement encouraging youth to smoke	Low	1,241 (94.44)	73 (5.56)	1.00	
	High	287 (45.63)	342 (54.37)	20.26 (15.26–26.89)	<0.001
Perception of cigarette ads message	Low	693 (81.91)	153 (18.09)	1.00	
	High	835 (76.12)	262 (23.88)	1.42 (1.13–1.78)	0.002
Attitude toward TAPS	Negative	1,458 (84.23)	273 (15.77)	1.00	
	Positive	70 (33.02)	142 (66.98)	10.84 (7.91–14.83)	<0.001
Exposure to cigarette ads	Low	1,047 (81.35)	240 (18.65)	1.00	
	High	481 (73.32)	175 (26.68)	1.59 (1.27–1.98)	<0.001
Exposure to cigarette marketing	Low	1,462 (80.20)	361 (19.80)	1.00	
	High	66 (55.00)	54 (45.00)	3.31 (2.27–4.83)	<0.001
Friend smoke	No	882 (94.33)	53 (5.67)	1.00	
	Yes	646 (64.09)	362 (35.91)	9.33 (6.87–12.66)	<0.001
Family smoke	No	833 (84.74)	150 (15.26)	1.00	
	Yes	695 (72.40)	265 (27.60)	2.12 (1.69–2.65)	<0.001
Susceptibility to smoke	No	1,493 (83.04)	305 (16.96)	1.00	
	Yes	35 (24.14)	110 (75.86)	15.38 (10.31–22.95)	<0.001
Knowledge of tobacco harm	Low	1,057 (81.62)	238 (18.38)	1.00	
	High	471 (72.69)	177 (27.31)	1.67 (1.33–2.08)	<0.001
Exposure to tobacco control education	No	440 (80.00)	110 (20.00)	1.00	0.359
	Yes	1,088 (78.10)	415 (21.36)	1.12 (0.88–1.43)	

### Multivariate analysis of smoking initiation

Table 4 shows the values adjusted for significant sociodemographic and social influence variables, perception about cigarette ads targeted at youths, attitude toward TAPS, and exposure to cigarette ads associated with smoking initiation in Model 9 (M9). The sociodemographic variables that related to smoking initiation were gender and age. Perception about cigarette ads targeted at youths and attitude toward TAPS were consistently associated with smoking initiation as well as with smoking friends and family. Pocket money was the confounding variable for the exposure to cigarette ads. When the variable of pocket money was not included in M9, the exposure to cigarette ads was associated with smoking initiation. Exposure to cigarette marketing and susceptibility had no association with smoking initiation when integrated into the models (M4 and M6).

**Table 4:** Multiple logistic regression: perception of cigarette ads, socio demography and social influence related to smoking initiation

Dependent variable: smoking initiation									

	Model 1	Model 2	Model 3	Model 4	Model 5	Model 6	Model 7	Model 8	Model 9
	
	OR (95% CI)	OR (95% CI)	OR (95% CI)	OR (95% CI)	OR (95% CI)	OR (95% CI)	OR (95% CI)	OR (95% CI)	OR (95% CI)
Sex									
Female	1					1	1	1	1
Male	3.67***					2.02***	2.02***	2.02***	2.00***
	(2.95–4.56)					(1.57–2.61)	(1.57–2.60)	(1.57–2.60)	(1.56–2.57)
Age (years)	1.10**					1.08*	1.07	1.08*	1.08*
	(1.03–1.18)					(1.00–1.16)	(1.00–1.16)	(1.00–1.16)	(1.01–1.17)
Pocket money (1,000 IDR)	1.02*					1.02	1.02	1.02	
	(1.00–1.04)					(1.00–1.04)	(1.00–1.04)	(1.00–1.04)	
Perception of cigarette advertisement targeted to youth									
Low		1							
High		1.23							
		(0.98–1.53)							
Perception of cigarette advertisement encouraging youth to smoke									
Low		1				1	1	1	1
High		4.64***				2.62***	2.59***	2.68***	2.70***
		(3.72–5.78)				(2.03–3.38)	(2.01–3.34)	(2.09–3.43)	(2.10–3.46)
Perception of cigarette advertising message									
Low		1							
High		0.97							
		(0.78–1.22)							
Attitude toward TAPS/tobacco advertising, promotion and sponsorship									
Negative			1			1	1	1	1
Positive			3.74***			1.50*	1.49*	1.51*	1.51*
			(2.79–5.01)			(1.08–2.09)	(1.07–2.07)	(1.09–2.11)	(1.08–2.09)
Exposure to cigarette ads									
Low				1		1	1	1	1
High				1.46***		1.26	1.25	1.25	1.27*
				(1.18–1.80)		(1.00–1.58)	(0.99–1.57)	(1.00–1.58)	(1.01–1.59)
Exposure to cigarette marketing and sponsorship									
Low				1		1			
High				1.43		0.74			
				(0.96–2.11)		(0.48–1.15)			
Friend smoke									
No					1	1	1	1	1
Yes					3.04***	1.66***	1.65***	1.68***	1.70***
					(2.42–3.82)	(1.28–2.16)	(1.27–2.14)	(1.29–2.17)	(1.32–2.21)
Family smoke									
No					1	1	1	1	1
Yes					1.36**	1.33*	1.32*	1.32*	1.31*
					(1.10–1.69)	(1.06–1.66)	(1.06–1.66)	(1.06–1.66)	(1.04–1.64)
Susceptibility to smoke									
No					1	1	1		
Yes					2.23***	1.31	1.28		
					(1.57–3.19)	(0.89–1.91)	(0.88–1.87)		
*N*	1,943	1,943	1,943	1,943	1,943	1,943	1,943	1,943	1,943
Pseudo R square	0.0719	0.0954	0.0343	0.00719	0.072	0.141	0.14	0.139	0.138
AIC	2,086	2033.4	2166.3	2228.9	2085.9	1945.4	1945.2	1944.8	1945.1
Degree of freedom	3	3	1	2	3	10	9	8	7

* p< 0.05

** p< 0.01

*** p< 0.001

### Multivariate analysis of smoking status

The adjusted analysis showing the similarity of the multivariate analysis of smoking status with smoking initiation is shown in Table 4. Perception about cigarette ads targeted at youths and attitude toward TAPS associated with smoking status are shown in Table 5. The sociodemographic variables gender, age, pocket money, and mother's education had an association with smoking status. The perception of cigarette ads targeted at youths, attitude toward TAPS, and susceptibility were consistently associated with smoking status and with smoking friends and family in a number of models (M6, 7, 8, 9, and 10). Pocket money and mother's education were always related to smoking status in several models (M7, 8, 9, 10). Exposure to cigarette ads and cigarette marketing were not related to smoking status after being integrated into the model.

**Table 5:** Multiple logistic regression: perception of cigarette ads, socio demography and psychosocial variables related to smoking status

Dependent variable: smoking status										

	Model A	Model B	Model C	Model D	Model E	Model F	Model G	Model H	Model I	Model J
	
	OR (95% CI)	OR (95% CI)	OR (95% CI)	OR (95% CI)	OR (95% CI)	OR (95% CI)	OR (95% CI)	OR (95% CI)	OR (95% CI)	OR (95% CI)
Sex										
Female	1					1	1	1	1	1
Male	10.3***					4.44***	4.44***	4.44***	4.44***	4.44***
	(7.67–13.9)					(3.07–6.42)	(3.07–6.42)	(3.07–6.42)	(3.07–6.41)	(3.07–6.41)
Age (years)	1.21***					1.23***	1.23***	1.24***	1.24***	1.25***
	(1.12–1.31)					(1.11–1.37)	(1.11–1.37)	(1.12–1.37)	(1.12–1.38)	(1.12–1.38)
Pocket Money (1,000 IDR)	1.04**					1.03*	1.03*	1.03*	1.03*	1.03*
	(1.01–1.06)					(1.00–1.06)	(1.00–1.06)	(1.00–1.06)	(1.00–1.06)	(1.01–1.06)
Fathers Education										
University	1					1				
Secondary	1.42*					1.13				
	(1.06–1.91)					(0.78–1.65)				
Mothers Education										
University	1					1	1	1	1	1
Secondary	1.42*					1.47	1.57**	1.58**	1.57**	1.56**
	(1.04–1.93)					(1.00–2.16)	(1.14–2.17)	(1.15–2.17)	(1.14–2.16)	(1.14–2.15)
Perception of cigarette advertisement targeted to youth										
Low		1				1	1			
High		1.54**				1.15	1.14			
		(1.17–2.03)				(0.84–1.58)	(0.83–1.57)			
Perception of cigarette advertisement encouraging youth to smoke										
Low		1				1	1	1	1	1
High		20.1***				7.33***	7.34***	7.48***	7.55***	7.63***
		(15.0–26.9)				(5.29–10.2)	(5.30–10.2)	(5.42–10.3)	(5.47–10.4)	(5.52–10.5)
Perception of cigarette advertising message										
Low		1								
High		0.79								
		(0.60–1.05)								
Attitude toward TAPS/tobacco advertising, promotion and sponsorship										
Negative			1			1	1	1	1	1
Positive			10.8***			3.26***	3.29***	3.29***	3.31***	3.32***
			(7.91–14.8)			(2.19–4.87)	(2.21–4.90)	(2.21–4.91)	(2.22–4.94)	(2.23–4.95)
Exposure to cigarette ads										
Low				1		1	1	1		
High				1.53***		1.18	1.18	1.2		
				(1.22–1.91)		(0.86–1.61)	(0.86–1.62)	(0.88–1.64)		
Exposure to cigarette marketing and sponsorship										
Low				1		1	1	1	1	
High				3.17***		1.45	1.44	1.46	1.47	
				(2.17–4.63)		(0.85–2.47)	(0.85–2.46)	(0.86–2.49)	(0.87–2.51)	
Friend smoke										
No					1	1	1	1	1	1
Yes					7.10***	2.37***	2.37***	2.38***	2.38***	2.41***
					(5.18–9.73)	(1.62–3.46)	(1.63–3.47)	(1.63–3.47)	(1.63–3.47)	(1.65–3.51)
Family smoke										
No					1	1	1	1	1	1
Yes					1.74***	1.57**	1.59**	1.61**	1.61**	1.60**
					(1.35–2.25)	(1.15–2.15)	(1.17–2.17)	(1.18–2.19)	(1.18–2.19)	(1.18–2.18)
Susceptibility to smoke										
No					1	1	1	1	1	1
Yes					10.1***	5.53***	5.49***	5.51***	5.52***	5.61***
					(6.57–15.4)	(3.39–9.02)	(3.37–8.94)	(3.38–8.98)	(3.39–9.00)	(3.44–9.13)
*N*	1,943	1,943	1,943	1,943	1,943	1,943	1,943	1,943	1,943	1,943
Pseudo R square	0.192	0.296	0.118	0.0245	0.223	0.446	0.446	0.446	0.445	0.444
AIC	1640.4	1427.9	1781.9	1972.2	1574.4	1144.4	1142.8	1141.5	1140.8	1140.8
Degree of freedom	5	3	1	2	3	13	12	11	10	9

* p< 0.05

** p< 0.01

*** p< 0.001

## Discussion

The prevalence of smoking among youths in this study was 21.36%, less than what has been found nationally (26.2%) (4) for the population aged 15–24 years. The percentage of male smokers in this study was 38.46%, which was also less than the national figure in 2012 (17) for 15–19-year-old male smokers (51.70%). This difference could be due to the definition of ‘smoker’. We defined a smoker as a person who smoked at least one cigarette per week, whereas in the Global Youth Tobacco Survey (17), a smoker is defined as a person who smokes at least once in 30 days.

How youths perceived cigarette advertisements targeted at youths was higher among those who had a high perception compared to those who had a low perception. These results indicated that the target of cigarette advertisement was young people, not as stated by the tobacco industry that TAPS is targeted at adults (10). Although more than half of young people in this study reported a low perception of cigarette advertisement encouraging youths to smoke, this result is alarming. The univariate analysis showed that somebody who has a high perception of cigarette advertisement encouraging youths to smoke is almost five times more likely to initiate smoking and 20 times more likely to become a smoker. Moreover, when adjusting for sociodemographic and social influences (friends and family smoke), the perception of cigarette advertisement encouraging youths to smoke was still significantly correlated with smoking initiation and smoking status. Youths who had a high perception of cigarette advertisement encouraging youths to smoke were 2.7 times more likely to initiate smoking and 7.7 times more likely to become a smoker. In a country where cigarette advertisement through electronic and printed media has not been banned and where the FCTC has not been ratified, this result could be used as the foundation to enforce government regulations of a TAPS ban and for signing the FCTC. According to the FCTC (9) article 13, all TAPS should be banned.

Only 11% of participants in this study had a positive attitude toward TAPS, but the univariate analysis showed that participants who had positive attitude toward TAPS were 3.7 times more likely to initiate smoking and 10.8 times more likely to become a smoker. This result echoed Su et al. who adopted the Theory of Planned Behavior (TPB). Su et al. reported that respondents who had favorable attitudes toward smoking on psychological aspect 3 to 7 were more likely to ever smoke and become regular smokers.

Exposure to cigarette ads was associated with smoking initiation and becoming a smoker in the univariate analysis. However, when adjusted with sociodemographic and social influences, exposure to cigarette ads only related to smoking initiation. Smoking initiation was one step to becoming a smoker (35, 37). As Hanewinkel et al. (35) reported, cigarette ads exposure was associated with ever having tried smoking (OR 1.97:1.40, 2.77) and current smoking (OR 2.90:1.48, 5.66). A longitudinal study carried out by Morgenstern et al. (23) supports the results of this study. In 30 months of Morgenstern's study, each additional 10 tobacco advertising contacts increased the adjusted relative risk for established smoking by 38% and for daily smoking by 30%. New findings in addiction show that as little as one cigarette can change the brain, modifying its neurons in a way that stimulates the craving to smoke (40, 41); this report warned that although it may ‘only’ initiate smoking, the risk is high for becoming a smoker.

In other studies (20, 31), smoking friends and family had an association with smoking behavior and mediating the association between exposure to cigarette ads and smoking behavior. After adjusting for social influence, we found that exposure to cigarette ads had a significant relationship to smoking initiation in this study. However, in relation to current smoking, a smoking friend or family also had a significant relationship in the final multivariate model, along with the perception of tobacco ads encouraging youths to smoke and the attitude toward TAPS.

This study has limitations. We did not study exposure to tobacco ads by asking participants how many times they had seen cigarette ads on TV, a movie, or on outdoor media, as in previous studies (30). The attitude toward TAPS was established by only two questions, and it was not based on any theory, such as TPB (28).

## Conclusions

This study revealed that cigarette advertising and promotional messages are targeted at youths. Gender, age, pocket money, and mother's education were the sociodemographic variables that had an association with smoking status. Perception of cigarette ads targeted at youths, attitude toward TAPS, and susceptibility were consistently associated with smoking status as were smoking friends and family. Pocket money and mother's education were always related to smoking status in several models. Although exposure to cigarette ads and cigarette marketing did not relate to smoking status after being integrated into the model, still the study found that cigarette advertising and promotional messages indeed are targeted at youths and their perception was strongly associated with smoking status. Regulations to ban TAPS in order to prevent youths from smoking should be applied rapidly in Indonesia.

## Ethics and consent process

The participating students and their parents or legal guardians gave signed informed consent to participate. Ethical approval to conduct the study was granted by the Medical and Health Research Ethics Committee Faculty of Medicine Universitas Gadjah Mada and Dr. Sardjito General Hospital.

## Paper Context

There are limited studies about youth perception of tobacco advertisement, promotions, and sponsorships (TAPS), particularly in a country like Indonesia, as the only Asia-Pacific country not having ratified FCTC, where TAPS is aggressive, and tobacco control remains in its infancy. It is interesting to understand more about how Indonesian youths perceive TAPS, which has become a major part of their environment, and the sociodemographic factors affecting smoking. It is hoped that this study will contribute toward Indonesia's tobacco control and prevention.
